# Nanoarchitectonics-Based Materials as a Promising Strategy in the Treatment of Endodontic Infections

**DOI:** 10.3390/pharmaceutics16060759

**Published:** 2024-06-04

**Authors:** Suli Xiao, Guanwen Sun, Shan Huang, Chen Lin, Yijun Li

**Affiliations:** 1 Department of Endodontics, Stomatological Hospital of Xiamen Medical College, Xiamen 361003, China; xiao1310508049@163.com; 2 Xiamen Key Laboratory of Stomatological Disease Diagnosis and Treatment, Xiamen 361003, China; 3 Department of Stomatology, Fujian Medical University Xiamen Humanity Hospital, Xiamen 361018, China; ydsgw@163.com; 4 Department of Stomatology, Zhongshan Hospital Affiliated to Xiamen University, Xiamen 361005, China; shanhuangfjmu@163.com

**Keywords:** nanomaterials, antimicrobial, endodontic infection, irrigant, intracanal medicament, sealer

## Abstract

Endodontic infections arise from the interactive activities of microbial communities colonizing in the intricate root canal system. The present study aims to update the latest knowledge of nanomaterials, their antimicrobial mechanisms, and their applications in endodontics. A detailed literature review of the current knowledge of nanomaterials used in endodontic applications was performed using the PubMed database. Antimicrobial nanomaterials with a small size, large specific surface area, and high chemical activity are introduced to act as irrigants, photosensitizer delivery systems, and medicaments, or to modify sealers. The application of nanomaterials in the endodontic field could enhance antimicrobial efficiency, increase dentin tubule penetration, and improve treatment outcomes. This study supports the potential of nanomaterials as a promising strategy in treating endodontic infections.

## 1. Introduction

Endodontic infection is an inflammatory disorder occurring in the root canal system and periradicular tissue and is characterized by bacterial infection or bone resorption. It stands as a primary contributor to tooth loss and is recognized as a risk factor associated with low-grade systemic inflammation, including cardiovascular diseases and diabetes mellitus [1,2,3].

The concept of endodontic treatment is based on the elimination of microbial biofilms as well as associated inflammation and damaged tissues from the root canal. The current treatment of endodontic infection relies on mechanical instrumentation; however, such a method alone cannot result in the complete cleaning of the complex root canal system due to the microbial complexity and the intricate anatomy of the root canal system. It is demonstrated that mechanical cleaning merely reduces the microbial count by 100- to 1000-fold [4]. Various irrigants like chlorhexidine (CHX) and sodium hypochlorite (NaClO) have been used as an adjunct to conventional mechanical instrumentation. Nevertheless, the combined approach of mechanical removal and chemical irrigation still possibly fails if microbes invade into the deeper dentin tubule and form a biofilm. The reason for this phenomenon might be due to the complex anatomy of the root canal system that is not easily accessible for antimicrobial agents. Moreover, these adjunctive methods are associated with undesired side effects, such as tooth staining, dry mouth, allergic reactions, and even inadvertent extrusion [5,6]. Based on prior systematic reviews, the overall success rate for initial endodontic treatment, following long-term observation, typically ranges from 60% to 80% [7,8]. However, the most recent retrospective study reveals a similar trend, indicating cumulative endodontic success rates of 93%, 85%, 81%, and 81% after 10, 20, 30, and 37 years, respectively, in the life table analysis [9]. Notably, the presence of large periapical lesions can significantly impact biological healing [10]. Specifically, for teeth with periapical lesions exceeding 5 mm, the success rates have been reported to vary between 38% and 80% [10,11]. For a long time, many studies have focused on how to improve the debridement of the root canal system and the outcome of root canal treatment [12,13].

Nanomaterials, characterized by their homogeneous particles within the 1–200 nm range, are increasingly designed to serve as antimicrobials or drug carriers in the anti-infection era. Antimicrobial nanoparticles can be developed from various materials, including metal or metal oxide particles, polymers, or graphene-based materials. These nanoparticles offer several advantages, making them promising candidates for combating microbial infections. One of the key advantages of antimicrobial nanoparticles is their high surface area-to-volume ratio, facilitating increased interaction with microorganisms. Moreover, the small size of nanoparticles enables them to penetrate microbial cell walls and biofilm matrices more effectively, which leads to better targeting and elimination of pathogens [14]. Additionally, nanoparticles possess multiple modes of antimicrobial action and have a lower possibility of inducing antimicrobial resistance [15]. In the realm of drug delivery, nanocarriers can protect conventional drugs from pH and enzymatic degradation within the harsh biofilm niche, while also leveraging these unique microenvironments for stimuli-responsive drug release [16,17]. This controlled-release capability helps optimize therapeutic effects and minimize potential side effects, making nanocarriers a valuable tool in drug delivery [18]. Given these benefits, researchers have shown a growing interest in introducing nanomaterials into the endodontic field. Here, we provide a comprehensive understanding of the potential applications of representative functional nanomaterials in the endodontic field.

The search for this review encompassed the PubMed database, focusing on publications spanning from January 2014 to January 2024. The search strategies were primarily crafted around the following keywords: (“endodontic infection” OR “irrigant” OR “irrigation” OR “intracanal medicament” OR “dressing” OR “sealer” OR “root canal obturation”) AND (“nanoparticle” OR “nanomaterial” OR “nanoplatform”). The scope encompassed both in vitro and in vivo experiments, encompassing both animal models and human studies. Notably, only articles published in English were considered eligible for inclusion. The exclusion criteria stringently excluded non-English papers, reviews, commentaries, letters to the editor, interviews, and updates.

## 2. Endodontic Pathogenic Species

Analogous to other oral infections, endodontic infection is caused by polymicrobial communities that act as a unit of pathogenicity. The polymicrobial communities are termed as highly organized biofilms surrounded by the self-produced extracellular polymeric substance (EPS) matrix. Previous studies have indicated that approximately 70% of endodontic infections originate from intracanal biofilms, which are responsible for subsequent inflammatory reactions, such as extreme pain, abscess formation, and cellulitis [19,20]. Meanwhile, endodontic failure is often reported to be associated with persistent biofilms in the intracanal environment [21].

**Figure 1:**
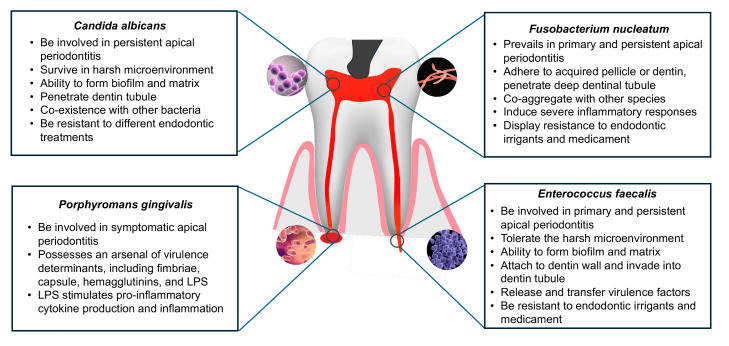
A schematic illustration of the main microbes in primary and secondary endodontic infections. *E. faecalis*, *P. gingivalis*, *F. nucleatum*, and *C. albicans* are prevalent in primary and secondary root canal infections. For *E. faecalis and C. albicans*, they can tolerate extreme conditions, form abundant biofilms, and are resistant to different endodontic treatments. With respect to *P. gingivalis* and *F. nucleatum*, their outstanding features are their ability to produce pro-inflammatory cytokines and induce inflammation.

Researchers have employed a diverse array of culture and molecular techniques to identify the microbiota present in endodontic infections. To date, studies have revealed the presence of over 500 bacterial species in these infections, with fungi, archaea, and viruses also being detected in association with apical periodontitis [22]. Notably, distinct types of microbiomes correspond to different clinical conditions of endodontic infection. Pulpitis is the infection of necrotic pulp following pulp exposure due to caries or trauma, which can be classified into irreversible pulpitis and reversible pulpitis. Among reversible pulpitis cases, Lactobacillus, Propionibacterium, Olsenella, and Actinomyces are the most prevalent genera [23]. In contrast to reversible pulpitis, there is a higher abundance of Lactobacillus in symptomatic irreversible pulpitis [23]. Another study collected root canal samples from adults and identified the enriched genera in irreversible pulpitis by using next-generation sequencing. The results showed that the most frequent genera were Veillonella, Streptococcus, Corynebacterium, Propionibacterium, and Porphyromonas [24]. Different detection methods may lead to different results of microbial composition in clinical samples. Checkerboard assay has shown that species from the genera Atopobium genomospecies, Pseudoramibacter alactolyticus, Streptococcus, Parvimonas micra, Fusobacterium nucleatum, and Veillonella are associated with irreversible pulpitis [25]. As for primary root canal infection, Fusobacterium species, Prevotella species, and other anaerobic species are the popular microbiota. Ferreira et al. identified that Fusobacterium species and Enterococcus species are resistant to different medications in primary root canal infections [26]. Obligatory anaerobic bacteria, Gram-positive facultative *Lactobacillus* species, and *Streptococcus* species are abundant in the dentin root canal walls of teeth with primary apical periodontitis [27]. Conversely, secondary root canal infections exhibit a less diverse microbiota compared to the primary form. Persistent root canal infections are typically polymicrobial biofilms composed of Enterococcus, Streptococcus, Peptostreptococcus, Prevotella, and Porphyromonas, all of which collaborate to survive [28]. Meanwhile, in teeth with post-treatment apical periodontitis, the anaerobe and facultative consist of the main communities [29]. A comprehensive understanding of microbial species and their virulence factor can help efficiently control the infection and impede the inflammatory process. Figure 1 lists some common microorganisms in endodontic infections.

### 2.1. Enterococcus faecalis (E. faecalis)

Though the detection rate of *E. faecalis* in clinical samples varies in previous publications, it is currently accepted that *E. faecalis* is the prominent pathogen in endodontic infection and the primary target in studies on antibacterial agents for endodontic treatment [30]. *E. faecalis* is related to different types of endodontic infections including primary and persistent apical periodontitis [31,32]. The detection rate of *E. faecalis* in failed root canal treatment cases is nine times higher than that in primary endodontic infections [33]. It is suggested that the presence of *E. faecalis* in saliva is a risk factor for root canal contamination with this pathogen [34]. Herein, we summarized the main virulence factors of *E. faecalis* in the process of endodontic infection.

Unlike the inherent oral cavity where the nutrition is rich, the root canal system is hypoxic and short of nutrition supply. Additionally, there is limited space for microbes to escape from medicaments in the root canal system. For microbial species residing in the dentin tubules of root canals, they should tolerate the harsh microenvironment to survive. Previous studies have demonstrated that *E. faecalis* biofilms can withstand harsh conditions including extreme alkaline pH, scarce nutrition, oxygen deprivation, and high salt concentrations [35,36]. Interestingly, *E. faecalis* is found to grow faster and form thicker biofilms under low-nutrition conditions than in nutrient-rich conditions [37]. Na^+^-ATPase activity, cell membrane surface hydrophobicity, and the upregulation of virulence and stress response genes contribute to *E. faecalis* survival in hostile conditions [38].

In addition to biofilm formation ability, *E. faecalis* can attach to dentin walls, invade dentin tubules, and remain viable within the tubule due to the presence of biofilm-associated pili (Ebps) and their collagen-binding protein (Ace). It is demonstrated that *E. faecalis* has the ability to invade the whole length of dentin tubules up to 1000 μm or close to the cementum [39]. The invasion depth depends on the survival environment, the shape of the dentin tubule opening, and the diameter of the dentin tubule. Therefore, eliminating *E. faecalis* from dentin tubules needs to be accomplished by a combination of mechanical instrumentation, irrigants, and antibacterial medicaments.

After invading dentin tubules, *E. faecalis* secretes its virulence factor to trigger inflammatory reactions in apical tissues. These virulence factors include lipoteichoic acids (LTAs), gelatinase, cytolysin, aggregation substances, and surface adhesins [40]. Importantly, *E. faecalis* has been shown to transfer virulence factors to certain species, thus increasing the pathogenicity of microbial communities [41]. The various stages of an endodontic infection, along with periapical inflammation, are related to these virulence factors. For instance, *E. faecalis* LTAs can induce macrophage autophagy and stimulate leukocytes to release inflammatory mediators to initiate inflammation [42]. The presence of gelatinase contributes to bone resorption of periradicular tissue and the degradation of dentin organic matrices [43]. Although certain bacterial byproducts might directly contribute to periradicular tissue damage, a substantial portion of the tissue injury is likely orchestrated by the host’s immune response to the bacterium and its derivatives. 

Bacterial biofilms of *E. faecalis* are highly resistant to current antiseptics due to the presence of the extracellular polymeric matrix produced by this microorganism. Furthermore, *E. faecalis* isolates show resistance toward common antibiotics like tetracycline, ciprofloxacin, and azithromycin [44,45]. Francisco et al. isolated *E. faecalis* strains from unsuccessful endodontic treatment and β-Lactamase activity was observed in 20% of the *E. faecalis* strains studied [46]. The resistance pattern of root canal *E. faecalis* can be acquired from the consumption of cheese and food-borne enterococci due to horizontal gene transfer [47].

The effectiveness of NaClO in eradicating *E. faecalis* biofilms within a short contact time of 1 min at a low concentration of 0.00625% is indeed remarkable [48]. It is demonstrated that antimicrobial action relies on contact time and concentration of use [49]. Its antimicrobial properties, coupled with the potential synergy when combined with ethylenediaminetetraacetic acid (EDTA), further enhance its efficacy [50]. While CHX also shows promise in eliminating *E. faecalis*, the comparison between the two irrigants remains inconclusive, likely due to variations in biofilm substrates, sampling procedures, and other methodological considerations.

### 2.2. Porphyromans gingivalis (P. gingivalis)

The lower oxygen tension in the apical third of the root canal favors the residence of strictly anaerobic bacteria over facultative and aerobic species [51]. *P. gingivalis* is a predominant periodontal bacterium and is also frequently detected in diseased apical tissue [29]. The prevalence of *P. gingivalis* detected in primary endodontic infection ranges from 5% to 65% due to different identification techniques among studies [52,53,54,55]. *P. gingivalis* plays a pivotal role in symptomatic apical periodontitis, especially acute apical abscesses [56], and it is associated with the pain, swelling, and purulent exudate associated with endodontic infections. A recent study has characterized the microbiota of teeth with endodontic treatment failure and found that *E. faecalis* and *P. gingivalis* are predominant in the microbiota of persistent infection, which is consistent with a clinical study reported by Barbosa-Ribeiro et al. [29]. This finding highlights the resistance profile of *P. gingivalis* during endodontic treatment.

*P. gingivalis* possesses an arsenal of virulence determinants, including fimbriae, capsule, hemagglutinins, lipopolysaccharide (LPS), and hydrolytic enzymes which establish its potency as one of the most pathogenic species in the mouth. Among these virulence factors, fimbriae are an essential factor for *P. gingivalis* colonization since they participate in bacterial adherence, coaggregation with other microorganisms, and invasion into targeted tissues, and can also trigger the release of pro-inflammatory cytokines [57]. Rôças et al. have demonstrated the distribution of *P. gingivalis* fimA genotypes in patients who suffer from primary endodontic infection [58]. LPS is another critical factor of *P. gingivalis*, which acts as a stimulant of the host’s innate immune response. It is demonstrated that the LPS level has a positive correlation with clinical symptoms and exudates from the canal systems. Veloso and his co-workers have investigated the macrophage polarization responses induced by *P. gingivalis* LPS *and Porphyromonas endodontalis* (*P. endodontalis*) LPS [59]. Their results suggest that *P. gingivalis* LPS instead of *P. endodontalis* LPS increases M1 macrophages and TNF-α, IL-1β, IL-6, and IL-12 cytokine secretion. 

*P. gingivalis* biofilms are susceptible to NaClO, as a contact time of 30 s can result in total killing [60]. However, CHX can effectively inhibit biofilm formation but cannot lead to complete elimination. Scanning electron microscope (SEM) observations have revealed that CHX only disrupts individual biofilm-forming *P. gingivalis* cells but does not destroy the biofilms [61].

### 2.3. Fusobacterium nucleatum (F. nucleatum) 

*F. nucleatum* is a Gram-negative obligate anaerobe which prevails in both primary and secondary root canal infections [62]. By using the Illumina Miseq platform, *F. nucleatum* was identified as a keystone taxon in root canals and periapical lesions of post-treatment endodontic infections [63]. Gomes et al. demonstrated that *F. nucleatum* is associated with specific signs and symptoms of endodontic origin such as pain, tenderness to percussion, wet canals, and purulent exudates [64]. *F. nucleatum* can adhere to acquired pellicle or dentin, penetrate deep dentinal tubules, and colonize at the apical area or even extraradicular sites, forming biofilms [65]. In addition, *F. nucleatum* exhibits a propensity for coaggregation with other bacterial species, often acting as a bridge between early and late colonizers within biofilms, thereby exerting robust synergistic effects both nutritionally and structurally with other bacteria [66]. The coaggregation ability of *F. nucleatum* relies on the presence of several adhesins, including Fap2, RadD, and aid. According to Villanueva et al., the co-existence of *F. nucleatum* and *Prevotella* species and *Porphyromonas* species is a risk factor for endodontic flare-ups and often results in a worsening situation of periapical inflammatory lesions [67].

LPS from the bacterial outer membrane is perhaps the most crucial virulence factor of *F. nucleatum*, constituting 4% of its cell wall. LPS purified from *F. nucleatum* was shown to induce a higher level of IL-1β and TNF-α released from macrophages when compared to *P. gingivalis* [68]. *F. nucleatum* has been demonstrated to adhere to and invade oral epithelial and endothelial cells, inducing severe inflammatory responses. Ran et al. identified the presence of *F. nucleatum* in periapical lesion and its products may induce the activation of nucleotide-binding oligomerization domain-like receptor protein 3 (NLRP3) and the absent in Melanoma 2 (AIM2) [69], two inflammasomes involved in the progression of apical periodontitis [70]. 

Despite its virulence factor, *F. nucleatum* also displays resistance toward irrigants and medicaments [26]. These characteristics contribute to its persistence in recurrent root canal infections. However, the contributing role of *F. nucleatum* in the development of endodontic infections is not extensively studied, and further related research is needed in the future.

Research investigating the effects of irrigation solution on *F. nucleatum* is limited. According to previous studies, it is demonstrated that *F. nucleatum* is sensitive to NaClO and CHX. When the biofilms were exposed to 0.5% or a higher concentration of NaOCl, no growth could be detected [71]. CHX at an exceeding concentration of 0.5% showed antibacterial effectiveness against *F. nucleatum* and CHX-treated cells presented an irregular cell wall [72,73].

### 2.4. Candida albicans (C. albicans)

Apart from bacteria, the contributing role of fungi in the pathogenesis of endodontic infection has become a newer research point. Not surprisingly, *C. albicans* occupies the largest proportion of all fungi detected from infected root canal. The estimated prevalence rate of *C. albicans* in endodontic infection ranges from 6% to 18% in previous reports [74,75]. The isolated rate of *C. albicans* in persistent endodontic infection is higher than in primary endodontic infection. The reason for the persistence of *C. albicans* lies in its ability to survive in diverse environmental conditions. For instance, it can tolerate alkaline pH environments so that it still survives after calcium hydroxide medicament. It seems that *C. albicans* is more resistant to calcium hydroxide compared to *E. faecalis* [76]. In addition, it also possesses the ability to endure starvation under anaerobic and aerobic conditions. 

In studying the contribution of *C. albicans* in the pathologies of endodontic infection, Sevilla et al. found that *C. albicans* penetrates dentin tubules and invades frank pulpal exposure through cracks and leakage [77]. Moreover, it has been demonstrated that *C. albicans* has a great affinity toward root canal filling materials, especially AH-Plus sealer [76]. The initial adhesion of *C. albican*s is an essential step for subsequent biofilm formation on root filling materials, which may induce endodontic therapy failure. *C. albican*s has the ability to form robust biofilms with yeasts and on different substrates. Alshanta et al. have shown that different clinical isolates of *C. albican*s can survive after 3% NaClO treatment and regrow to form levels that are comparable with untreated biofilms [78]. The biofilm lifestyle boosts the tolerance of *C. albican*s to host defense and antifungal agents, ultimately aiding its persistence within the root canal. 

Recently, the co-existence of *C. albican*s and oral bacteria has been well documented in various oral diseases [79,80]. The interaction between *C. albican*s and *E. faecalis* is increasingly evident. Siqueria and Rocas have found that *E. faecalis* adheres to yeast and hyphal cells of *C. albicans* in infected tooth root canals as well as in dentinal tubules by using a scanning electron microscope for observations [27]. The cross-kingdom biofilm formed by *C. albican*s and *E. faecalis* presents as thicker and denser when compared to a single counterpart and exhibits higher tolerance to common chemical irrigants such as NaClO and CHX and even antimicrobial photodynamic therapy [81]. An in vivo experiment has demonstrated that co-inoculation of *E. faecalis* and *C. albicans* has significantly increased the extent of periapical lesions and upregulated inflammatory cytokines in periapical lesions [82]. It is reasonable to assume that the co-existence of *C. albican*s and *E. faecali*s contributes to the progression of endodontic infections.

Owing to the broad antimicrobial spectrum of NaClO and CHX, it is reported that both irrigants could exert antifungal effects on *C. albicans*. Earlier published articles have indicated that CHX has superior ability in disinfecting *C. albicans* [83]. Other irrigation solutions like QMix, MTAD, and Tetraclean also have antifungal activity [84].

### 2.5. Endodontic Microorganisms and Systemic Diseases

Previous studies have indicated a potential relation between systemic diseases and the pathogenesis of apical periodontitis [85]. Specifically, apical periodontitis is closely tied to an excessive microbial load and heightened systemic inflammation, ultimately contributing to the development of generalized low-grade inflammation within the human body. It has been demonstrated that certain oral microorganisms and their metabolic byproducts are potentially linked to systemic diseases, such as cardiovascular disease, gastrointestinal and colorectal cancer, diabetes, and Alzheimer’s disease [86,87]. Among these oral microbes, *P. gingivalis* infection stands out as having a particularly high correlation with systemic diseases. This bacterium has the capacity to directly invade the epithelium, endothelial cells, and subepithelial tissues. Furthermore, a persistent local infection caused by *P. gingivalis* prompts the upregulation of inflammatory cascades, potentially contributing to the pathogenesis of systemic disorders. Additionally, specific virulence factors of *P. gingivalis*, such as gingipains and outer membrane vesicles (OMVs), play crucial roles in the progression of local diseases, enabling the bacterium to invade distant tissues and participate in the initiation or advancement of systemic diseases [88]. Emerging evidence has also revealed a correlation, but not necessarily a causation, between *F. nucleatum* and systemic diseases. Akin to *P. gingivalis*, *F. nucleatum* can also gain access to the circulatory system, leading to transient bacteremia triggered by routine activities. This frequent entry into the circulation allows the bacteria to reach organs throughout the body, contributing to local pathogenesis [89]. In addition, *F. nucleatum* is a potent trigger of inflammatory cytokines, causing persistent local infection that upregulates inflammatory cascades [90,91]. *F. nucleatum* accelerates the progression of various systemic diseases by upregulating the expression of diverse TLRs. Lastly, specific toxins produced by *F. nucleatum*, notably FadA, Fap2, and LPS, play a crucial role in inducing local diseases [92,93].

## 3. Nanomaterials Used in Endodontics and Their Antimicrobial Mechanisms

### 3.1. Silver Nanoparticles (AgNPs)

Among nanoparticles, silver nanoparticles (AgNPs) have been the most extensively studied and used in various fields such as dentistry. The reason behind the dramatic attention paid to AgNPs is due to their broad antimicrobial spectrum against various biofilms [94,95,96]. Therefore, the direct application of AgNPs would act as a disinfectant against pathogenic microorganisms in the oral cavity. The sizes of AgNPs reported in previous studies range from 2 nm to a maximum of 120 nm. Transmission electron microscope images have indicated that AgNPs are nearly spherical (Figure S1).

**Figure 2:**
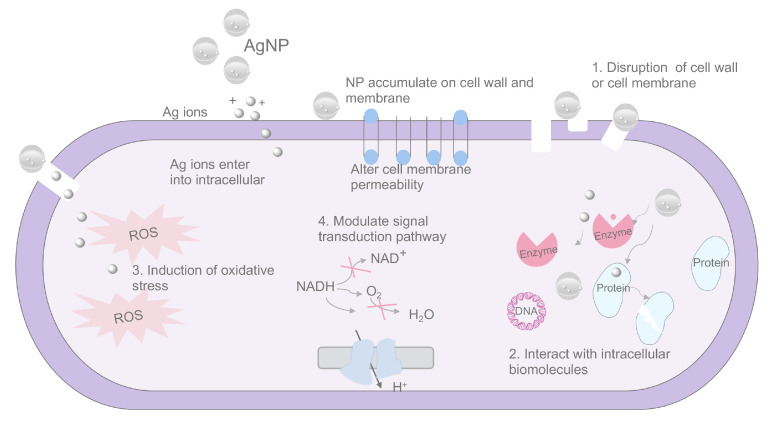
The proposed antimicrobial mechanisms of silver nanoparticles. The released Ag ions from AgNPs have great affinity to cell walls and membranes, altering the permeability of the cytoplasmic membrane and ultimately leading to bacterial envelope destruction. Meanwhile, AgNPs can interact with intracellular structures and biomolecules to induce the dysfunction of microbes. Additionally, Ag ions and AgNPs have the ability to induce excessive ROS to trigger cellular death. AgNPs can modulate signal transduction pathways in bacteria.

The main mechanisms behind microbial killing of AgNPs have been depicted in previous reviews [97,98,99]. The antimicrobial actions of AgNPs are related to four well-identified mechanisms (Figure 2): (1) The released silver ions are essential during the antimicrobial activity of AgNPs. Silver ions have a great affinity to cell walls and membranes, altering the permeability of the cytoplasmic membrane and ultimately leading to bacterial envelope destruction. (2) Once AgNPs enter cells, they can interact with intracellular structures and biomolecules to induce dysfunction. (3) Ag ions and AgNPs have the ability to produce excessive ROS which are responsible for cellular toxicity. (4) AgNPs can modulate signal transduction pathways in bacteria.

In addition to exerting a killing effect on pathogens, AgNPs can also impact biofilm formation from various aspects. The EPS matrix serves as an inherent physical barrier in bacterial biofilms, aiding bacteria in resisting adverse external stimuli and drug assaults. AgNPs have been reported to enhance the permeability of bacterial biofilms, facilitating increased diffusion of nanoparticles through the EPS matrix [100,101]. Moreover, studies suggest that AgNPs possess anti-quorum sensing properties, enabling them to disrupt signaling pathways within biofilms [102,103]. This interference with quorum sensing mechanisms can impede the coordinated behavior of bacteria within the biofilm, potentially affecting their ability to form and maintain the biofilm structure. By targeting these essential pathways, AgNPs contribute to the disruption of biofilm formation and function.

The effects of AgNPs on endodontic pathogens have undergone a series of investigations. AgNPs have a highly bactericidal action on *E. faecalis*, a prominent endodontic bacterium, with an MIC value of 30 μg/mL and a comparable effect with 0.2% CHX. They have also shown satisfactory antibacterial and antibiofilm activity against beta-lactamase-resistant *E. faecalis* [94]. Transcription analysis has revealed that the expression levels of genes associated with membrane transport and signal transduction and metabolism in *E. faecalis* after exposure to AgNPs were significantly affected [104]. In addition to presenting a killing effect on *E. faecalis*, AgNPs have shown potential for the elimination of *P. gingivalis*, *F. nucleatum*, and *C. albicans* biofilms [105,106,107]. Comparative studies have also proved that AgNPs have a superior ability in tacking *C. albicans* biofilm compared to common irrigants like NaClO or CHX [108,109]. Oncu et al. have established *C. albicans* biofilms on dentin blocks for seven days and subsequently treated these biofilms with AgNPs, 5.25% NaClO, and 2% CHX. SEM observation indicated a notable reduction in the number of planktonic cells in the specimens treated with AgNPs compared to the other groups. Moreover, the MIC analysis further validated the superior antifungal effect of AgNPs among the tested agents [108]. In another comparative study conducted by Panpaliya et al., the antifungal properties of 100 μg/mL CHX were juxtaposed against AgNPs. Their findings revealed that AgNPs exhibited a greater capacity to kill *C. albicans* compared to CHX, thereby establishing the superior antifungal potential of silver nanoparticles [109]. These studies highlight the antimicrobial potential of AgNPs in the endodontic field.

### 3.2. Chitosan Nanoparticles

Chitosan (CS), a biopolymer from chitin after alkaline deacetylation, has been widely used in wound healing, antimicrobials, tissue engineering, and drug delivery. It possesses three active functional groups, namely, the amino group at the C-6 position and the primary and secondary hydroxyl groups at C-6 and C-3 positions. These functional groups confer on CS special properties including antimicrobial activity, antifungal activity, and biocompatible features. The inhibitory effect of CS on pathogens is linked with the degree of deacetylation, positive charge density, molecular weight, microbe type, environmental pH, and temperature [110]. It has been demonstrated that CS nanoparticles (CSNPs) harbor higher antimicrobial capacity than CS due to their higher surface-to-volume ratio [111]. CSNPs can be synthesized through ionotropic gelation, microemulsion, emulsification solvent diffusion, polyelectrolyte complex methods, and so on. And they can be processed into different forms like gel, film, paste, microsphere, and microparticle. 

**Figure 3:**
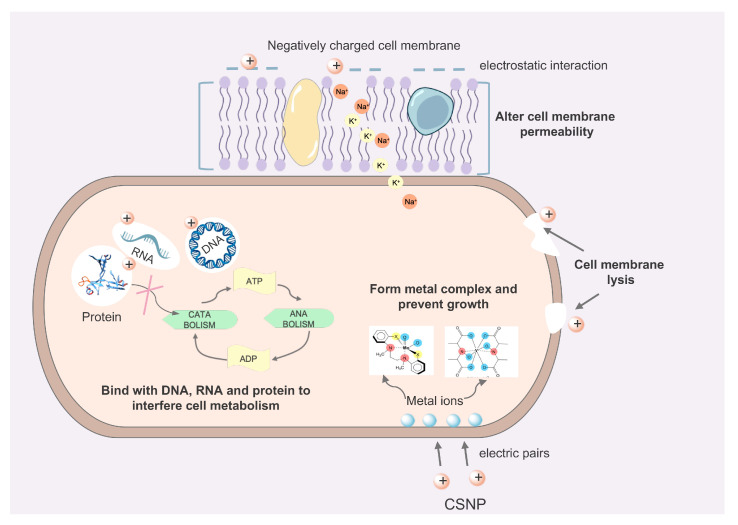
The proposed antimicrobial mechanisms of chitosan nanoparticles. The positive charge of the CSNPs allows them to interact with negatively charged cell membranes through electrostatic interactions and to elicit cell membrane permeability alteration and cell membrane lysis. In addition, CSNPs can bind with DNA, RNA, and protein to exert an inhibitory effect. Furthermore, CSNPs donate electron pairs to the metal ions on the bacterial surface to form complexes and prevent the growth of microorganisms.

Several antimicrobial mechanisms of CS have been proposed previously (Figure 3), including the following: (1) the positively charged CS molecule interacts with the negatively charged cell membrane through electrostatic interaction and elicits cell membrane permeability alteration and cell membrane lysis, (2) CS can bind with DNA, RNA, or other biomolecules to interrupt microbes’ metabolism, and (3) CS donates electron pairs to the metal ions on the bacterial surface to form complexes and prevent the growth of microorganisms [112]. Owing to its surface charge, CS is expected to interact electrostatically with negatively charged biofilm components such as EPS, protein, and DNA to exert an inhibitory effect. Regarding its effects on biofilms, it has been proven that CS can be used for (1) the inhibition of adhesion stages, (2) the prevention of biofilm formation, (3) the disruption of mature biofilm structures, and (4) interference with quorum sensing pathway. 

Regarding the application of CSNPs in endodontics, Kishen et al. were the first team to evaluate the potential of CSNPs in root canal disinfection. CSNPs can penetrate the complexities of the root canal and dentinal tubules, thus eliminating microorganisms based on their concentration and time-dependent property, even after 3 months [113]. Meanwhile, the antimicrobial effect of CSNPs is not influenced by the presence of inhibitors such as pulpal remnants and bovine serum albumin [114].

### 3.3. Quaternary Ammonium Compounds (QACs)

Quaternary ammonium compounds (QACs), classified as cationic surfactants, are nitrogen-containing compounds wherein the nitrogen atom forms covalent bonds with four distinct groups. The historical genesis of QACs as preservatives and disinfectants dates back to the 1930s. Their introduction to the dental field occurred in the 1970s, developed initially as mouthwashes to impede oral biofilm formation. Presently, antimicrobial QACs encompass monomeric QACs, gemini QACs, polymeric QACs, bola-amphiphilic salts, and those featuring two or more polar groups [115]. QACs exhibit notable efficacy against oral microorganisms such as *S. mutans*, *S. gordonii*, *C. albicans*, and *E. faecalis* [116,117], while also demonstrating potential in mitigating the development of drug resistance compared to antibiotics.

**Figure 4:**
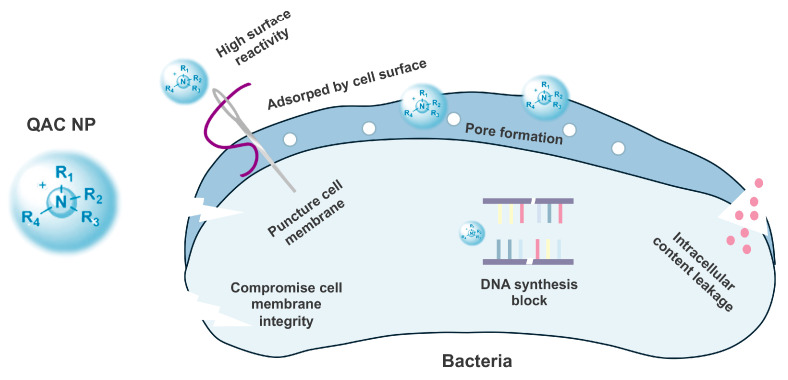
The proposed antimicrobial mechanisms of quaternary ammonium compounds.

Despite QACs showcasing remarkable biofilm eradication capabilities by disrupting microbial cells, impeding bacterial adhesion, and influencing biofilm formation, the intricate details of their killing mechanisms remain incompletely elucidated (Figure 4). The cationic nature of QACs imparts high surface reactivity, facilitating adsorption by the cell surface. The prevailing hypothesis posits that the antimicrobial mechanism primarily relies on electrostatic interactions between the positively charged QACs and the negatively charged cell surface [118]. Upon binding to the cell membrane, the hydrophobic tail of QACs inserts into the bacterial membrane, akin to a needle puncturing a balloon [119]. This insertion results in pore formation, compromised cell integrity, leakage of low-molecular components, and eventual microbial death. Many QACs possess membrane-damaging properties, and some are reported to interfere with DNA replication and synthesis, as well as suppress FtsZ polymerization and cell division [120].

Several factors influence the antimicrobial effects of QACs, including hydrophobicity, alkyl chain length, charge density, and structural features. Enhanced hydrophobicity has been correlated with increased antimicrobial efficacy, but an excessive increase may lead to diminished antimicrobial activity and heightened hemolytic activity [121]. Longer alkyl chains in QACs enhance penetration of the cell membrane and interaction with intracellular biomolecules due to weaker hydrophobic interactions. However, beyond a certain threshold length (>12), the alkyl chain may bend, impeding interactions with the cell membrane [122]. Structural rigidity or the presence of active alkaloids in QAC skeletons is associated with a greater propensity to exhibit antimicrobial activity.

### 3.4. Graphene-Based Materials

Graphene is a single layer of carbon atoms arranged in a two-dimensional honeycomb lattice. Graphene-based materials include diverse materials comprising pristine graphene, few-layer graphite platelets, and oxidized or reduced forms of them, which can be synthesized by bottom-up and top-down methods [123]. Graphene and its derivatives hold great promise in the medical field, especially in terms of antimicrobial applications, due to its unique properties, including a very high value of a specific surface area, inherent antimicrobial properties, exceptional electrical conductivity, high thermal conductivity, and excellent surface modifiability. Accumulating studies have demonstrated that graphene-based materials display excellent antimicrobial activity. For instance, Cao et al. reported that graphene oxide flakes exhibit antimicrobial effects on Gram-positive and Gram-negative bacteria and fungi both in planktonic and biofilm states [124]. Another study showed that vertically aligned graphene impacts bacterial survival and the mechanical stability of biofilms [125]. Scanning electron microscope images have revealed that exposed edges of vertically aligned graphene flakes penetrate the bacterial membrane and drain the intracellular content. Additionally, bacteria are not able to develop resistance to this killing mechanism over multiple exposures [126].

Previous reviews have provided insights into the antimicrobial mechanisms of graphene-based materials, which can be classified into several categories [127,128]. The nanoknife action of graphene plays a pivotal role in its antimicrobial impact. The sharp edges of graphene, resembling blades or cutters, are primarily responsible for physically puncturing cell membranes. This action leads to the leakage of cellular contents and eventual cell death [129]. Additionally, graphene-based materials exhibit the ability to induce oxidative stress upon interaction with microbial cells. The burst of oxidative stress can occur through either a reactive oxygen species (ROS)-dependent or a ROS-independent pathway [130]. This oxidative stress results in cellular dysfunction and inactivation. Moreover, the unique flexibility conferred by the thinner structure of graphene allows it to act as a barrier. This property enables graphene to wrap around and isolate bacteria from the surrounding environment [128]. The flexibility of graphene, akin to a protective shield, contributes to its antimicrobial effectiveness by impeding the normal function of microbial cells. 

### 3.5. Metal Oxide Nanoparticles

Metal oxides are crystalline solids that contain a metal cation and an oxide anion. They are currently undergoing extensive investigations as promising antimicrobial agents. Their appeal lies in their relatively low toxicity toward human cells, cost-effectiveness, and size-dependent efficacy against a broad spectrum of bacteria [131]. Zinc oxide nanoparticles (ZnO-NPs), titanium oxide (TiO_2_) nanoparticles, Fe_3_O_4_, and copper nanoparticles (CuNPs) are being widely investigated in dentistry. The sizes of metal oxides in previous reports range from 10 nm to 70 nm [132,133]. SEM images have shown that metal oxide nanoparticles are predominantly spherical-shaped (Figure S2). Moreover, these metal oxides exhibit the ability to prevent biofilm formation and even eliminate spores, further enhancing their suitability for applications in the fabric, skincare, biomedical, and food-additive industries [134]. Djearamane et al. synthesized ZnO nanoparticles and explored the antibacterial effects of different concentrations of ZnO-NPs on *E. faecalis*. It has been revealed that ZnO-NPs have an inhibitory effect on *E. faecalis* and cause the loss of integrity of cell membranes and the distortion of bacterial cells [135]. CuNPs possess contact antibacterial ability and can alter DNA or protein synthesis, inactivate their enzymes, and promote the generation of hydrogen peroxide. Rojas and his co-workers found that CuNPs have an immediate action and over-time effect on multispecies biofilms. This ability allows CuNPs to play a role in intracanal medication [136]. The definitive mechanism of metal oxides is not clear, but the proposed mechanisms are (1) the release of metal ions, (2) ROS generation, (3) the binding of metal oxides to the bacterial membrane, and (4) cellular internalization.

## 4. The Application of Nanomaterials Used in Endodontics

**Figure 5:**
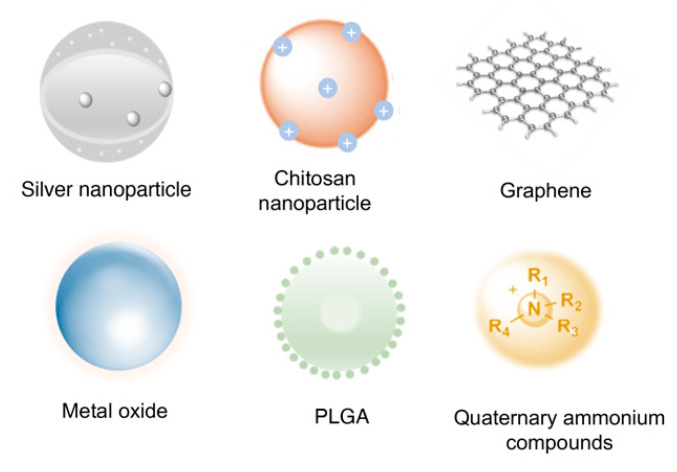
Representative functional nanomaterials used in endodontics.

**Figure 6:**
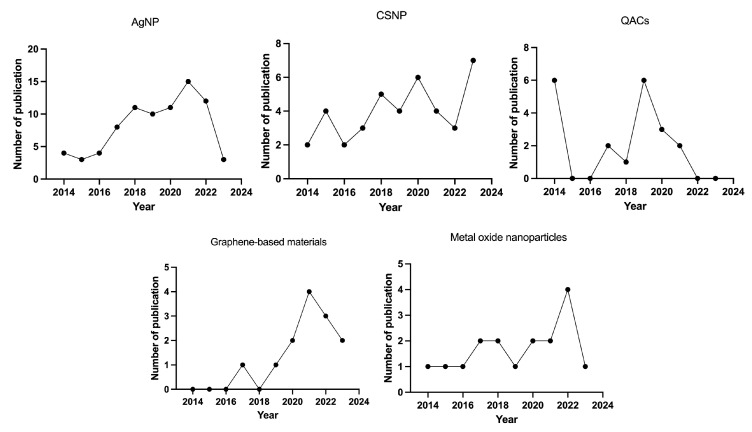
The number of publications related to the different materials used in endodontics in this review.

Owing to the outstanding features of nanomaterials, more and more studies have tried to use nanomaterials to improve the treatment outcome of endodontic infection (Figure 5). The number of publications related to the different materials used in endodontics are shown in Figure 6.

### 4.1. Nanomaterials Act as Irrigants

The substantial surface area-to-mass ratio and increased chemical reactivity of nanoparticles endow them with an enhanced ability to interact with microbes, surpassing that of conventional antiseptics. Selected examples from the published literature are presented in Table 1. In the realm of root canal disinfection, nanoparticles, particularly AgNPs, have been explored for their potential as irrigants. In a study by Rodrigues et al., a AgNP irrigant, 2.5% NaClO, and 2% CHX were tested against *E. faecalis* biofilms and infected dentinal tubules [137]. Treatment at various intervals (5, 15, and 30 min) revealed that the AgNP irrigant was less effective than NaClO in disrupting *E. faecalis* biofilm formation and eradicating biofilms from dentinal tubules. This outcome aligns with Wu et al.’s earlier study, where a 0.1% AgNP solution, applied for 2 min, failed to disrupt the biofilm structure [138]. The limited effect of AgNPs as an endodontic irrigant may be attributed to the short contact time with the root canal and the protective role of the biofilm matrix. Seeking to enhance root canal disinfection by using AgNPs, Afhkami et al. explored different activation systems for AgNP solutions and assessed their impact on *E. faecalis* biofilm elimination [139]. Their findings suggested that activation methods such as photon-induced photoacoustic streaming or passive ultrasonic irrigation could potentiate the AgNP irrigant’s ability to disrupt *E. faecalis* biofilm formation. Ioannidis et al. used GO to act as a matrix that can compensate for the lack of stability and aggregation of sole AgNP dispersion and is expected to have an enhanced and synergistic antimicrobial action. In their study, they developed a novel tooth model possessing lateral canals and evaluated the antimicrobial effects of different irrigants. The antimicrobial efficacy of Ag-GO in lateral canals was superior to that of 1% NaClO, 2% CHX, 17% EDTA, and sterile saline. Another study also illustrated a similar finding that incorporating graphene into AgNPs showed strong antibacterial properties, as effective as 3% NaClO, but with less cytotoxic effects on bones and soft tissues [140].

Irrigation with AgNPs may influence the biochemical properties of root dentin. Syringe irrigation with a standard AgNP solution for 15 min decreased the microhardness of root canal dentin in the apical and coronal thirds [141]. Conversely, Jowkar et al. demonstrated that using AgNP nanoparticles as the final irrigant improved the fracture resistance of teeth with previous endodontic treatment [142]. Another study reported a similar finding that irrigation with AgNPs caused little to no alterations in the mechanical properties of dentin and resin cement in different root canal thirds [143]. The conflicting results among these studies may stem from variations in AgNP particle size, surface properties, contact time, and the maturation degree of the biofilm. 

In addition to AgNPs, CSNPs have also been explored as an endodontic irrigation solution. Root canal irrigation with CS has shown promise in improving smear layer removal due to its ability to chelate various metallic ions and inorganic components within the smear layer [144]. The efficacy of a CSNP solution in disrupting microbial biofilm within the root canal system has been demonstrated, with its effect being comparable to that of 3% NaClO [145]. Traditional root canal irrigants have been associated with a decrease in the biomechanical properties of dentin walls, making the tooth more susceptible to fractures. This poses a challenge for regenerative endodontic procedures, especially in diseased teeth with thinner dentin walls and shorter root lengths. Pascale et al. assessed the effects of CS irrigation on the biochemical and biomechanical properties of dentin [146]. Their findings indicated that CS treatment induced a higher modulus of elasticity and lower proteolytic activity. Moreover, CS-treated EDTA-demineralized dentin specimens exhibited significantly less biodegradation. This finding is consistent with the results of an experiment by Ozlek et al., demonstrating that CS irrigation enhances the dislocation resistance of an MTA–resin hybrid root canal sealer compared to EDTA and saline irrigation [147]. Additionally, final irrigation with CSNPs allows for the release of proteins including transforming growth factor-beta 1 (TGF-β1), vascular endothelial growth factor (VEGF), and dentin sialoprotein (DSP) immersed in the dentin matrix, suggesting that CSNPs can act as a protector of the exposed organic fraction on the root canal surface [148]. These results underscore the multifunctional capabilities of CS as an endodontic irrigant, showcasing its potential benefits in eradicating biofilms and preserving dentin integrity.

**Table 1:** Nanomaterials’ function as irrigants for endodontic irrigation.

NP	Group	Substrate	Microbe	Detection Method	Other Detection	Conclusion	Reference
AgNPs	G 1: 94 ppm AgNP solution G 2: 2.5% NaOCl G 3: 2% CHX	Bovine dentin blocks	*E. faecalis*	Live/dead technique	NA	AgNP irrigant was not as effective against *E. faecalis* as solutions commonly used in root canal treatment. NaOCl is appropriate as an irrigant because it is effective in disrupting biofilm formation and eliminating bacteria in biofilms and dentinal tubules.	[137]
AgNPs	G 1: 0.1% AgNP solution G 2: 2% NaOCl G 3: sterile saline	Dentin sections	*E. faecalis*	Live/dead technique	NA	The findings from this study suggest that the antibiofilm efficacy of AgNPs depends on the mode of application. AgNPs as a medicament and not as an irrigant showed potential to eliminate residual bacterial biofilms during root canal disinfection.	[138]
AgNPs	G 1: AgNP solution G 2: AgNPs + ICG-aPDT G 3: AgNPs + PIPS G 4: AgNPs + MDA G 5: AgNPs + PUI G 6: 2.5% NaOCl G 7: no intervention	Human single-root teeth	*E. faecalis*	Counting of forming units	NA	Activation with PUI and PIPS enhanced the efficacy of the AgNP irrigating solution for the elimination of *E. faecalis* from the root canal system.	[139]
AgNPs Graphene	G 1: AgNPs + graphene G 2: 3% NaOCl G 3: saline	Mandibular and premolar	*E. faecalis*	Counting of forming units	NA	Within the confines of this study, graphene–silver composite nanoparticles demonstrated maximum antimicrobial effectiveness against *E. Faecalis* bacteria.	[140]
ZnONPs AgNPs Imidazolium-based silver (Im-AgNPs)	G 1: 0.1% ZnONPs G 2: AgNPs G 3: Im-AgNPs G 4: 2.5%NaOCl G 5: normal saline	Mandibular and premolar	NA	NA	Dentin microhardness	The irrigants containing Im-AgNPs and ZnONPs significantly enhanced the root dentin microhardness. However, the use of AgNPs resulted in decreased microhardness.	[141]
AgNPs TiO_2_ NPs ZNO-NPs	G 1: normal saline G 2: 2% CHX G 3: 17% EDTA + 2.5% NaOCl G 4: 17% EDTA + 0.1% AgNPs G 5: 17% EDTA + 0.1% TiO_2_ G 6: 17% EDTA + 0.1% ZNO-NPs	Single-root premolar teeth	NA	NA	Fracture resistance	The final irrigation of root canals with nanoparticles enhanced the fracture resistance of the endodontically treated roots. The lowest fracture resistance value was observed for NaOCl.	[142]
AgNPs	G 1: distilled water G 2: 5.25% NaClO G 3: 25% polyacrylic acid G 4: 2% CHX G 5: 23 ppm AgNPs	Single-root human teeth	NA	NA	Hardness and elastic modulus	Silver nanoparticle application is a viable option for the irrigation of the intraradicular dentin previously achieved through the cementation process of glass-fiber posts.	[143]
CSNPs	G 1: 0.2% CSNPs solution G 2: 17% EDTA G 3: solution	Mandibular and premolar	NA	NA	Dislocation resistance	This study provides the first evidence that chitosan irrigation improves the dislocation resistance of an MTA–resin hybrid root canal sealer, compared to EDTA and saline irrigation.	[147]
EDTA Chitosan CSNPs	G 1: 0.2% CSNPs solution G 2: 17% EDTA G 3: solution	Dentin	NA	NA	Root dentin matrix of proteins Chemical change	Proteins can be released from dentin via EDTA, CS, and CSnp conditioning. Raman spectra revealed changes in the inorganic content of the root dentin after chelation. Furthermore, the use of CSnps facilitated the preservation of the organic content.	[148]
QIS	G 1: saline G 2: 6% NaOCl G 3: 6% NaOCl + 2% CHX G 4: 2% CHX, G 5:0.5% k21/E G 6: 1% k21/E	Single-root teeth	*E. faecalis*	Live/dead staining	Alizarin-red with sortase-A active sites using Schrödinger docking Root dentin substrate	The current investigation showed that a new concentration of a 0.5% k21/E irrigant has the ability to reduce and disrupt *E. faecalis* biofilm formation, which can subsequently improve root canal treatment outcomes.	[149]
QIS	G 1: 6% NaOCl + 2% CHX G 2: 3.5% QIS G 3: 2% QIS G 4: sterile saline	Single-root teeth	*E. faecalis*	Live/dead staining Forming unit assay	Sealer penetration Cytotoxicity	The novel QIS root canal irrigant achieved optimum antimicrobial protection inside the root canals compared to 6% NaOCl alone and 6% NaOCl + 2% CHX.	[150]
QIS	G 1: saline G 2: 0.5% K21 G 3: 1% K21	Dentin slabs	*E. faecalis*	Live/dead staining Scanning electron microscopy	Mitochondrial morphology Pro-inflammatory cytokine level Cell viability Wound healing	Altogether, our study reports the antimicrobial and reparative efficacy of a k21 disinfectant which is a proof of concept for enhanced killing of bacteria across the root dentin.	[151]

NA: not applicable; NaOCl: sodium hypochlorite; ICG-aPDT: indocyanine green–antimicrobial photodynamic therapy; PIPS: photon-induced photoacoustic streaming; PUI: passive ultrasonic irrigation; MDA: manual dynamic activation; CHX: chlorhexidine; MTA: mineral trioxide aggregate; EDTA: ethylenediaminetetraacetic acid; QIS: quaternary ammonium silane.

The aforementioned studies have elucidated the promising avenues that nanomaterials offer in enhancing antimicrobial effectiveness and optimizing root canal disinfection. Beyond merely augmenting antimicrobial capabilities, these nanomaterials also demonstrate potential in eliminating smear layers, suppressing MMP activity, and maintaining the integrity of dentin collagen compared to NaClO. Nevertheless, the efficacy of nanomaterials in assisting irrigation procedures is contingent upon factors such as nanoparticle size, concentration, and duration of contact.

Daood and co-workers have conducted an array of experiments to investigate the potential role of QAC as an endodontic irrigant [149,150,151]. They used a sol–gel reaction to synthesize a novel quaternary ammonium silane called K21. As a contact-killing material, K21 exerts antimicrobial activity through its long, lipophilic –C18H37 alkyl chain penetrating the bacterial membranes, causing autolysis and cell death [152]. K21 causes a significant reduction in live bacteria and the destruction of *E. faecalis* biofilms in a concentration-dependent manner, with a concentration of 3.5% nearly reaching complete cell death [150]. Microscope observations have revealed cell membrane destruction, cell integrity loss, and cytoplasmic leakage of bacterial cells following K21 treatment [153]. Moreover, the crust formation due to K21 irrigation remained active after seven days and prevented bacteria from regrowing. In addition to broad-spectrum antibacterial performance with very low cytotoxicity, the K21 molecule elicits anti-MMP activity [154], which prevents further destruction of host tissues. This is because of the binding of positive ammonium ions on negative glutamic acid residues, blocking the active site of MMP and preventing enzymatic degradation of dentin. Moreover, K21 has the ability to initiate a series of immune responses for active host defense and tissue repair [151]. K21 is demonstrated to reduce chemotaxis to injured tissues by impairing macrophage sensitization to chemokines and modulating macrophage functional polarization into the M2 phenotype. An in vivo study also indicated that K21 has the potential to greatly improve wound healing and to be used as a wound care product [153]. The effect of K21 irrigation on some surface properties of root canals was also investigated. Aided irrigation with K21 resulted in higher wettability and surface free energy of root canal dentin, which may favor the long-term success of root canal therapy. NaClO irrigation is reported to impact dentin collagen fiber and induce substantial changes in intratubular glycosaminoglycans [155,156]. In contrast to NaClO irrigation, dentin specimens with K21 irrigation showed integrated, parallel, and dense collagen fibers with evident cross-binding. These findings suggest that K21 is a biocompatible, efficient, and safe irrigant for endodontic therapy.

### 4.2. Nanomaterial-Based Antimicrobial Photodynamic Therapy for Root Canal Disinfection

Antimicrobial photodynamic therapy (aPDT) is an alternative treatment option for the treatment of oral infections which relies on the photochemical interaction of a light source, a photosensitizer, and oxygen [157]. The core mechanism of aPDT lies in the generation of reactive oxygen species (ROS), including hydroxyl radicals, hydrogen peroxide, and singlet oxygen, which effectively eliminate pathogens. It possesses several advantages over common antibiotics including a broad antimicrobial spectrum, minimal side effects, and low possibility of resistance development. Previous studies have demonstrated the potential of aPDT in endodontics, both in in vitro and in vivo models, as well as in randomized clinical trials [158,159]. However, the complex structure of the root canal system and the hydrophobic nature of photosensitizers often hinder the photodynamic effect in eradicating endodontic biofilms. To address this challenge, nanomaterials have been employed to create nanocarriers that enhance the properties of photosensitizers, thereby boosting their antimicrobial effect.

Rose Bengal (RB), an established xanthene photosensitizer, exhibits remarkable photoactivation capabilities against a range of oral microbes, attributed to its high quantum yield of ^1^O_2_. However, its limited use in dentistry is hampered by several drawbacks, including poor photostability, hydrophobicity, and an anionic charge [160]. To address these challenges, Shrestha et al. conjugated RB with CSNPs, creating a novel compound, CSRBnp, and explored its potential in treating root canal infections. CSRBnp, upon photoactivation, demonstrated significant antibacterial and antibiofilm activity against *E. faecalis* [161]. Furthermore, this compound, in combination with light irradiation, effectively eliminated multispecies biofilms, inactivated endotoxins, and resisted biological degradation [162,163]. The mechanisms underlying the enhanced antimicrobial activity of CSRBnp involve improved the binding of RB to bacterial cells and a reduced release rate of ^1^O_2_. The limited light penetration into the root canal dentin tubule weakens the effect of aPDT on endodontic biofilms. Upconversion nanoparticles (UCNPs) have been developed in the hope of absorbing near-infrared (NIR) light sources to emit visible light to trigger PSs for generating ROS. Zong et al. developed a novel triple-layered core–shell nanostructure UCNP@SiO_2_/methylene blue (MB) @quaternized chitosan (QCh) and evaluated the photodynamic potential of this new synthesized compound [164]. They proved that UCNP@SiO_2_/MB@QCh could stick close to the bacteria and generate abundant ROS to destroy *E. faecalis* biofilms.

The introduction of nanomaterials into the aPDT field holds significant potential for enhancing the delivery of photosensitizers (PSs) and the subsequent outcomes of aPDT. The encapsulation or integration of PSs into nanomaterials has led to several advancements, including heightened bioavailability and solubility, minimized side effects, controlled-release mechanisms, targeted delivery to infection sites, and bolstered therapeutic efficacy of PSs. While numerous studies have reported promising results with nanomaterial-based PSs, there is still a pressing need for a comprehensive analysis of newly designed PSs and in vivo models to accurately demonstrate their actual therapeutic effectiveness.

### 4.3. Nanomaterials Act as Intracanal Medicaments

Studies have shown that 40–60% of microbes remain alive within the root canal system after chemical irrigations [165,166,167]. Antimicrobial medicament during visits aims to prevent bacterial proliferation, eliminate surviving bacteria, and reduce microbial penetration resulting from microleakage. However, recent high-quality reviews claim that using an intracanal dressing does not provide extra benefit in teeth with apical periodontitis, though previous research has focused on additional intracanal medication after debridement of infected teeth. Meanwhile, in cases that have an increased probability of endodontic failure and a low healing rate, it is suggested to use intracanal medicament.

While Ca(OH)_2_ has shown promising results as an intracanal medicament, its effectiveness is undermined by the natural buffering ability of dentin and hydroxyapatite against alkaline substances, which diminishes the antimicrobial capacity of Ca(OH)_2_. Furthermore, it has been established that Ca(OH)_2_ can only penetrate up to 126 μm into dentin tubules, whereas bacteria can infiltrate as deep as 400 μm [168]. There is also a concern that Ca(OH)_2_ paste may compromise the microhardness of dentin. To address these limitations, the use of nanoparticles has been suggested either as an alternative to Ca(OH)_2_ or to enhance its antimicrobial properties. Nano-sized medicaments can ensure an optimal therapeutic index through their effect on microorganisms both at the sub-cellular and molecular levels. Selected examples from the published literature are presented in Table 2.

The utilization of AgNPs as an intracanal medicament has exhibited noteworthy efficacy in reducing *E. faecalis* biofilm cells, surpassing the antibiofilm capabilities of Ca(OH)_2_ under the same placement duration [169]. Furthermore, dentin discs treated for 7 days with concentrations of AgNP gel (0.01–0.02%) allowed more than 90% of DPSC cells to attach after 24 h, suggesting that AgNP gel may represent a promising future candidate for clinical use in regenerative endodontics [170]. This result is in line with another report, which suggests that the application of AgNPs alone or in combination with Ca(OH)_2_ holds great promise as an intracanal medicament in root canal treatments and endodontic regeneration [171]. However, the sustained and controlled release of AgNPs cannot be easily achieved and this behavior may sometimes compromise their efficiency as a medicament [172]. Liu et al. addressed the challenge of sustained effectiveness by formulating AgNP poloxamer thermoreversible gels (AgPLs) [173]. Their findings indicate that AgPLs exhibit stronger abilities against biofilms, greater penetration depth, and less tooth discoloration compared to Ca(OH)_2_ as well as low cytotoxicity toward human periodontal gingival fibroblasts. The positive results of AgPLs may be due to the sustained release of Ag+ ions from AgNPs -PL, which is expected to prolong and sustain the antimicrobial action. CSNPs have also been evaluated as an intracanal medicament, with a result of appreciable antimicrobial properties and fracture resistance when compared to nano-Ca(OH)_2_ [174].

Another prevalent strategy involves integrating nanoparticles with Ca(OH)_2_ paste. These nanoparticles range from silver (AgNPs), copper nanoparticles, and CSNPs to zinc oxide (ZnO) nanoparticles. A comparative study used CSNPs and ethanolic propolis extract (EPE) to incorporate them into Ca(OH)_2_ paste to kill 21-day-old *E. faecalis* single biofilms and 48 h intraoral biofilms. This finding suggests that incorporating CSNPs into a Ca(OH)_2_-based paste has the potential to increase its antibacterial activity and inhibit bacterial recolonization on root canal dentin; meanwhile, the Ca(OH)_2_/EPE paste was not able to maintain its antibacterial efficacy over time [175]. Teja et al. explored the additive effects of various nanoparticles combined with Ca(OH)_2_ on *E. faecalis* biofilms, concluding that AgNP incorporation into Ca(OH)_2_ showcased superior efficacy [176]. Accumulating studies have also demonstrated that AgNPs mixed with Ca(OH)_2_ present more pronounced antibiofilm effects against *E. faecalis*, *C. albicans*, *F. nucleatum*, and even multispecies biofilms compared to Ca(OH)_2_ [177,178,179,180]. The combination of NPs with Ca(OH)_2_ can bring about additive or synergistic effects on root canal disinfection.

Alternatively, nanoparticles can serve as innovative carriers for delivering Ca(OH)_2_ into the root canal system. Polymer nanoparticles, such as PLGA, CS, and gelatin, are among the top choices for this purpose. For example, Ca(OH)_2_ has been successfully encapsulated within PLGA nanoparticles, resulting in a significantly enhanced penetration depth and area within dentinal tubules, according to confocal laser scanning microscopy assessments [181]. This increase in penetration is likely due to the small size of Ca(OH)_2_ nanoparticles (below 200 μm), much less than the diameter of a dentin tubule (2400–4280 μm). Moreover, Ca(OH)_2_ nanoparticles have shown a capacity for prolonged and steady drug release at high concentrations, as opposed to free Ca(OH)_2_. For nanocarrier delivery, Ca(OH)_2_ presented improved antibiofilm capacity against multispecies biofilms consisting of *E. faecalis*, *C. albicans*, and *S. gordoni* [182]. The recruitment of mesenchymal stem cells and their osteogenic potential is a determinant in the regeneration of periapical tissues. Thus, the osteoblastic cell responses of endodontic materials have been widely explored. Ca(OH)_2_-loaded PLGA NPs might impact the differentiation and maturation of osteoclasts, which is essential for the resorption process [183]. In another study, Ca(OH)_2_ encapsulation with CS/gelatin nanoparticles achieved controlled, sustained release of calcium ions [184]. Sustained and controlled release is crucial for the therapeutic success of intracanal medicaments. Therefore, a suitable carrier may ensure high penetration of Ca(OH)_2_ through the tubules and slow and steady release of calcium and hydroxyl ions.

**Table 2:** Functional nanomaterials used as intracanal medicaments.

NP	Mode	Group	Microbe	Placement Duration	Other Detection	Conclusion	Reference
NCH Nanochitosan	Alone	G 1: calcium hydroxide G 2: NCH G 3: chitosan G 4: nanochitosan	*E. faecalis*	1 month	Depth of penetration Fracture resistance	Calcium hydroxide showed a limited depth of penetration and it significantly reduced the fracture resistance of teeth. Chitosan showed greater fracture resistance compared to all other groups, but had a limited depth of penetration. Both nanoforms showed superior penetration into the dentinal tubules and appreciable antibacterial efficacy.	[174]
AgNPs	Alone	G 1: PL alone G 2: Ca(OH)_2_ paste G 3: 16 μg/mL AgNPs -PL G 4: 32 μg/mL AgNPs PL	*E. faecalis*	9 days	Dentinal tubule penetration Color evaluation	The prepared gels exhibited advantages of sustained release of Ag^+^ and strong antibiofilm effects against *E. faecalis* for 9 days.	[173]
AgNPs	Alone	G 1: Ca(OH)_2_ G 2: AgNPs G 3: AgNPs + Ca(OH)_2_ G 4: positive control G 5: negative control	*E. faecalis*	7 days 14 days	NA	It was concluded that the antibacterial effect of AgNPs was lower than that of Ca(OH)_2_ or of the combination of both materials.	[172]
AgNPs	Alone	G 1: no treatment G 2: 0.01% AgNPs G 3: 0.015% AgNPs G 4: 0.02% AgNPs G 5: Ca(OH)_2_	NA	NA	Cytotoxicity Cell viability Cell proliferation	The cytotoxicity and proliferation of dental pulp stem cells in response to the AgNP gel were comparable to those resulting from calcium hydroxide.	[170]
Chitosan AgNPs	Mixed	G 1: Ca(OH)_2_ G 2: Ca(OH)_2_ + 2% CHX gel G 3: Ca(OH)_2_ + 2% chitosan gel G 4: Ca(OH)_2_ + 0.02% AgNP gel G 5: Ca(OH)_2_ + bioactive glass	*E. faecalis*	7 days	NA	The combination with calcium hydroxide showed higher antibacterial efficacy than when used alone. Calcium hydroxide with bioactive glass showed better antimicrobial efficiency against *E. faecalis* when compared to other combinations of calcium hydroxide.	[176]
AgNPs	Mixed	G 1: saline solution G 2: Ca(OH)_2_ G 3: Ca(OH)_2_ + AgNPs G 4: 2% CHX gel G 5: Ca(OH)_2_ + 2% CHX gel	Multispecies biofilm	7 days	NA	The present study put forth the potential use of AgNPs mixed with Ca(OH)_2_ or CHX on multispecies (*Enterococcus faecalis*, *Streptococcus mutans*, *Lactobacillus acidophilus,* and *Actinomyces naeslundii*) biofilms over 1- and 7-day application periods.	[177]
AgNPs	Mixed	G 1: sterile water G 2: Ca(OH)_2_ G 3: Ca(OH)_2_ + AgNPs	*E. faecalis*	7 days	NA	This study highlighted the potential advantage of using a mixture of Ca(OH)_2_ and nanosilver as an intracanal medicament.	[169]
AgNPs	Mixed	G 1: cells alone G 2: Ca(OH)_2_ G 3: 0.04% AgNPs G 4: 0.06% AgNPs G 5: Ca(OH)_2_ + 0.03% AgNPs G 6: Ca(OH)_2_ + 0.04% AgNPs G 7: Ca(OH)_2_ + 0.06% AgNPs G 8: triple antibiotic paste	NA	NA	Cell morphology and attachment Cell proliferation Wound healing Osteogenic differentiation	These results highlight the potential application of AgNPs combined with Ca(OH)_2_ or alone as a biomaterial for various clinical applications, such as intracanal medicaments in root canal treatments and endodontic regeneration.	[171]
AgNPs	Mixed	G 1: triple antibiotic paste (TAP) G 2: Ca(OH)_2_ G 3: AgNPs G 4: Ca(OH)_2_ + AgNPs	*E. faecalis*	2 and 4 weeks	NA	The mixture of Ca(OH)_2_ + AgNPs showed a high antibiofilm effect that was not significantly different from 1 mg/mL TAP. Furthermore, long-term contact between intracanal medicaments and bacterial cells achieved significant antibiofilm efficacy.	[178]
AgNPs	Mixed	G 1: non-infected dentin samples G 2: infected dentin samples G 3: Ca(OH)_2_ G 4: 2% CHX G 5: AgNPs + Ca(OH)_2_	*C. albicans*	7 days	NA	It can be concluded that a combination of Ca(OH)_2_ and 0.04% AgNPs showed the most effective antibiofilm activity against *C. albicans* biofilms.	[179]
AgNPs	Mixed	G 1: TAP G 2: Ca(OH)_2_ G 3: 0.04% AgNPs G 4: 0.06% AgNPs G 5: Ca(OH)_2_ + 0.03% AgNPs G 6: Ca(OH)_2_ + 0.04% AgNPs G 7: Ca(OH)_2_ + 0.06% AgNPs	*F. nucleatum*	7 days 14 days	NA	The results of this study suggest a promising alternative of AgNPs as an intracanal medicament, especially in combination with Ca(OH)_2_.	[180]
CSNPs EPE	Mixed	G 1: distilled water G 2: Ca(OH)_2_ G 3: Ca(OH)_2_ + CSNPs G 4: Ca(OH)_2_ + EPE	*E. faecalis* Intraoral biofilm	7 days 14 days	NA	Incorporating CSNPs into a Ca(OH)_2_-based paste has the potential to increase its antibacterial activity and inhibit bacterial recolonization on root canal dentin after endodontic therapy. The Ca(OH)_2_/EPE paste was not able to maintain its antibacterial efficacy over time.	[175]
PLGA	Carrier	G 1: Ca(OH)_2_ G 2: Ca(OH)_2_ -PLGA	NA	NA	Depth and area of penetration	Ca(OH)_2_-loaded PLGA NPs were successfully optimized and characterized. The NPs exhibited a prolonged drug release profile and superior penetration inside dentinal tubules of extracted teeth when compared to Ca(OH)_2_.	[181]
PLGA	Carrier	G 1: saline solution G 2: chlorhexidine G 3: Ca(OH)_2_ G 4: Ca(OH)_2_-PLGA	Multispecies biofilm	7 days	NA	The CH-loaded PLGA NPs possessed a greater antimicrobial property against the multispecies biofilms of *E. faecalis*, *S. gordonii,* and *C. albicans* compared to conventional Ca(OH)_2_.	[182]
PLGA	Carrier	G 1: control medium G 2: Ca(OH)_2_-PLGA G 3: Ca(OH)_2_	NA	NA	Cytotoxicity Osteoclast formation	Ca(OH)_2_-loaded PLGA nanoparticles have the potential to inhibit osteoclast differentiation more effectively compared to Ca(OH)_2_ nanoparticles.	[183]

NCH: nano calcium hydroxide; PL: poloxamer; NA: not applicable; CHX: chlorhexidine; TAP: triple antibiotic paste; CSNP: chitosan nanoparticle; EPE: ethanolic propolis extract; PLGA: poly(lactic-co-glycolic).

Overall, the reported evidence demonstrates that nanoparticles, especially AgNPs, show potential as an intracanal medicament when in combination with Ca(OH)_2_. The addition of nanoparticles into original medicaments could improve antimicrobial outcomes, shorten medicament time, and increase dentin tubule penetration. However, there still exists some drawbacks of AgNPs that should be taken into consideration, such as tooth discoloration and toxic effects on surrounding cells.

### 4.4. Modification of Sealers by Nanomaterials

In the final step of root canal treatment, root canal obturation is crucial for preventing bacterial reinfection and facilitating host repair and tissue regeneration. While gutta-percha is the primary obturating material, it alone may not fully fill the root canal space or adhere to its walls. Previous research highlights the significance of microleakages between the root canal sealer, canal wall, and dentinal tubules, as they can lead to microbial regrowth and recontamination, ultimately influencing treatment success [185]. The properties of root canal sealers play a vital role in achieving effective three-dimensional root canal obturation. The ideal sealers should meet the following requirements according to Grossman [186]: an excellent seal ability, dimensional stability, a slow setting time to ensure sufficient working time, insolubility to tissue fluids, adequate adhesion with canal walls, and biocompatibility. However, none of the current available sealers fully satisfy all requirements, especially in the antimicrobial aspect. Most endodontic sealers have mild antimicrobial capacity and even diminish after setting. In addition, microorganisms have a great affinity for root canal filling like gutta-percha and subsequently form a biofilm to trigger infection again. Therefore, how to enhance the long-term antimicrobial effectiveness of endodontic sealers has become a concern and research topic in recent years. Selected examples from the published literature are presented in Table 3.

Exploring the incorporation of nanoparticles in endodontic sealers is an intriguing avenue, potentially decreasing further growth of the remaining microorganisms and improving treatment outcomes. The incorporation of QACs into endodontic sealers was the most investigated method in previous studies. Beyth et al. first formulated the incorporation of QACs (quaternary ammonium polyethyleneimine) into an epoxy-based resin sealer and evaluated the antibacterial efficacy, biocompatibility, and physical and chemical properties of the novel sealer [187]. The results indicated that this newly developed sealer exhibits a potent and prolonged antibacterial activity, coupled with adequate physical properties. Barros et al. conducted experiments to assess the potential of a novel sealer that incorporates quaternary ammonium polyethyleneimine (QPEI) into AH plus or pulp canal sealer EWT (PCS) [188,189,190]. They evaluated the antimicrobial properties, physicochemical properties, mechanical properties, and biocompatibility with the periapical tissue at different concentrations. The addition of QPEI to PCS led to a greater reduction in bacterial counts compared to AH plus, attributed to the interference of other components in AH plus that affect charge availability The incorporation of QPEI into PCS made it more hydrophilic, enhancing contact and penetration into biofilm structures and favoring bacterial elimination, without influencing physicochemical and mechanical properties such as solubility, flow, compressive strength, and dimensional stability compared to the original formulation. Additionally, the incorporation did not induce toxicity to osteoblastic or osteoclastic cells but had the capacity to modulate the proliferation and differentiation of bone cells [189]. In line with the results observed by Barros et al., Baras and his co-workers also found that the addition of dimethylaminohexadecyl methacrylate (DMAHDM) into AH plus shows potent antibacterial effects on *E. faecalis* biofilms [191]. Besides killing *E. faecalis* on the dentin surface, the novel sealer developed by their team could regenerate mineral loss during irrigation procedures and increase dentin hardness due to the incorporation of amorphous calcium phosphate (NACP). These consistent findings suggest that the incorporation of QACs into sealers results in strong and long-term antibacterial activities, without compromising the physical and mechanical properties. The addition of dimethylaminododecyl methacrylate (DMADDM) into different dental materials could improve antibacterial effects against pathogens and biofilms. Another group has aimed to modify EndoREZ with DMADDM and magnetic nanoparticles (MNP) [191]. The modification could lead to good sealing performance, penetration, and long-term antibacterial property.

**Table 3:** The incorporation of nanomaterials into sealers for root canal obturation.

NP	Group	Sealer Type	Microorganism	Detection Method	Other Detection	Conclusion	Reference
QPEI	G 1: RCS G 2: 1.5% QPEI + RCS	RCS	*E. faecalis*	Counting of forming units	Solubility Thermal analysis Flow assay Cytotoxicity assessment	As shown in the present study, affixation of polycationic antimicrobial nanoparticles in an endodontic sealer revealed long-lasting antimicrobial potency, providing an effective antimicrobial alternative.	[187]
QAS	G 1: AH plus G 2: 2% QAS + AH plus G 3: 4% QAS + AH plus G 4: 8% QAS + AH plus	AH plus	*E. faecalis*	DCT Live/dead staining	NA	The incorporation of QAES into epoxy resin-based AH plus may be a promising approach for controlling endodontic infection at the time of canal filling and preventing subsequent reinfection.	[192]
QPEI	G 1: control G 2: AH plus G 3: 1% QPEI + AH plus G 4: 2% QPEI + AH plus G 5: PCS G 6: 1% QPEI + PCS G 7: 2% QPEI + PCS	AH plus Pulp canal sealer (PCS)	*E. faecalis*	DCT	Setting time Flow test Solubility Apparent porosity Dimensional change Wettability Zeta potential Compressive strength	The incorporation of QPEI nanoparticles can improve the long-term antibacterial activity of pulp canal sealer EWT without relevant changes in physicochemical and mechanical properties.	[190]
QPEI	G 1: control G 2: 1% QPEI + AH plus/PCS G 3: 2% QPEI + AH plus/PCS G 4: 5% QPEI + AH plus/PCS G 5: 10% QPEI + AH plus/PCS	AH plus PCS	NA	NA	ALP (osteoblastic cells) and TRAP (osteoclastic cells) activities Apoptosis Intracellular signaling pathways	QPEI nanoparticles, at 2%, did not affect cell behavior. However, the incorporation of 2% QPEI particles into AH plus and PCS modulated the proliferation and differentiation of bone cells, depending on the sealer and the cell type, without increasing the sealers’ cytotoxicity.	[189]
QPEI	G 1: AH plus G 2: 2% QPEI + AH plus G 3: PCS G 4: 2% QPEI + PCS	AH plus PCS	*E. faecalis*	DCT CV staining	NA	The addition of QPEI nanoparticles improved the killing ability of PCS against biofilms of both *E. faecalis* strains and the effects of AH plus on the biomass of biofilms from the ATCC strain.	[188]
DAMHDM Sliver NACP	G 1: AH plus G 2: BTH + 40% glass + DAMHDM + Ag + NACP G 3: BTH + 30% glass + DAMHDM + Ag + NACP G 4: BTH + 20% glass + DAMHDM + Ag + NACP G 5: BTH + 10% glass + DAMHDM + Ag + NACP	BTH	*E. faecalis*	Live/dead staining	Flow properties Film thickness Ca and P ion release Dentin hardness	This new sealer with highly desirable antibacterial and remineralization properties showed promise in increasing the success rate of endodontic therapy and strengthening the tooth root structures.	[191]
AgNPs	G 1: AH plus G 2: AgNPs + AH plus	AH plus	Saliva	NA	NA	Silver nanoparticles used in tested concentrations did not improve the bacterial leakage resistance of the AH plus sealer.	[193]
ZnO nanoparticles	G 1: Grossman sealer G 2: 25% ZNO-NPs + Grossman sealer G 3: 50% ZNO-NPs + Grossman sealer G 4: 75% ZNO-NPs + Grossman sealer G 5: 100% ZNO- NPs + Grossman sealer	Grossman sealer	NA	NA	Setting time Flow Solubility Dimensional change	ZnO-Nps decreased the setting time and dimensional changes characteristic of Grossman sealer.	[194]
DMADMM MNPs	G 1: EndoREZ G 2: iRoot SP G 3: EndoREZ + 2.5% DMADDM and 1% MNPs	EndoREZ	*E. faecalis*	CFU Live/dead staining	Dentin tubule penetration Cytotoxicity	Overall, the current study found that compared with iRoot SP, the modified root canal sealer had good sealing performance, penetration, and long-term antibacterial property in the single-cone technique.	[195]

NA: not applicable; QPEI: quaternary ammonium polyethyleneimine; QAS: quaternary ammonium epoxysilicate; PCS: pulp canal sealer; DCT: direct contact test; NACP: calcium phosphate nanoparticle; DAMHDM: dimethylaminohexadecyl methacrylate; DMADMM: dimethylaminododecyl methacrylate; MNPs: magnetic nanoparticles. Strong and long-term antibacterial activities are shown, without compromising the physical and mechanical properties. The addition of dimethylaminododecyl methacrylate (DMADDM) into different dental materials can improve antibacterial effects against pathogens and biofilms. Another group has aimed to modify EndoREZ with DMADDM and magnetic nanoparticles (MNPs) [195]. The modification can lead to good sealing performance, penetration, and long-term antibacterial properties.

Apart from QACs, metal and metal oxides have been used to improve the antimicrobial efficiency of current sealers. For instance, Rücker et al. produced silver core–shell nanoparticles and demonstrated that 10 wt% concentration promoted a reduction in *E. faecalis* viability in the biofilm that was maintained for up to 9 months of storage [196]. Additionally, up to 10%wt concentration of this novel sealer still maintained the necessary physicochemical properties and did not induce cytotoxicity to fibroblasts. However, the AgNPs used did not improve the bacterial leakage resistance of the AH plus sealer after 3 months of observation [193]. Nanoparticles of ZnO were also added to the original sealer and this modification inhibited biofilm formation within the sealer– dentin interface, reduced cytotoxicity, and improved physicochemical properties [195]. It was concluded that the addition of ZnO NPs into a calcium hydroxide-based sealer shows better antibiofilm efficacy than CSNPs against *E. faecalis*. Additionally, another report demonstrated that the incorporation of ZnO nanoparticles into a ZOE-based sealer enhances the sealing ability and is suitable for preventing leakage in root canal therapy [133]. Apart from nanomaterial-based nanoparticles, calcium silicate-based bioactive sealers are now increasingly studied for root canal fillings due to their numerous biological advantages including alkalinity, calcium ion release, apatite-forming ability, and mineralization induction [197]. They are available in two forms: powder/liquid or pre-mixed ready-to-use syringes. The current calcium silicate-based sealers on the market include Bio-C Sealer, TotalFill BC Sealer, EndoSequence BC Sealer HiFlow, and iRoot SP. Compared to conventional sealers, calcium silicate-based sealers present greater and longer-lasting antimicrobial capacity [198]. Common sealers like AH plus only possess enough antimicrobial activity during the first day and dramatically reduce in effectiveness over longer times. This property is not beneficial for the long-term outcome of root canal obturation. Meanwhile, for calcium silicate-based sealers, it is reported that they could still exhibit antimicrobial ability after one or two weeks of placement [199]. The excellent antimicrobial action of calcium silicate-based sealers is attributed to their ability to maintain high pH and release hydroxyl ions consistently. In addition to adequate antimicrobial capacity, calcium silicate-based sealers can boost potential in cell proliferation, migration, and differentiation, which is indispensable for apical tissue healing [200]. The incorporation of nanomaterials into conventional sealers significantly boosts the antimicrobial effectiveness and prolongs the antimicrobial effect. In addition to antimicrobial efficacy, nanomaterial modifications have been used to improve the physicochemical properties of endodontic sealers, such as sealing ability, bioactivity, and radiopacity. While achievements of nanomaterials have been reported, it is worth noting that sometimes, nanomaterial modification could comprise the physicochemical features of the original sealer. Therefore, attention should be given to undesirable effects and endeavors should be made to avoid adverse effects.

### 4.5. Hydrogel-Based Materials in Endodontic Treatment

Regenerative endodontic treatment (RET) has been proposed a promising alternative treatment for immature teeth in cases of necrotic pulp/apical periodontitis due to traumatic injuries or caries lesions [201]. The process of RET involves utilizing a temporary scaffold composed of biomacromolecules and functional stem cells to replace the inflamed dental pulp and promote the generation of vital pulp-like tissues. Advancements in nanotechnology have spurred the development of hydrogels as scaffolds for RET, leveraging their unique properties such as tunable physiomechanical properties and remarkable biocompatibility [202]. Specifically, injectable hydrogels have been tailored to mimic the soft tissue environment, fostering a physiologically conducive niche for cell growth. Furthermore, their injectable nature significantly simplifies clinical procedures, reducing chair time and enhancing patient comfort. By taking advantage of injectable hydrogel, Astudillo-Ortiz et al. investigated the potential of an injectable hyaluronic acid-based (HA) hydrogel system which consisted of aldehyde-modified hydrogel (a-HA), hydrazide-modified hydrogel (ADH), and platelet lysate (PL) for endodontic regeneration [203]. The porous hydrogel favors the migration, nutrition, and proliferation of dental pulp cells, ultimately being replaced by an extracellular matrix derived from these cells following degradation.

Beyond promoting tissue regeneration, hydrogels have been ingeniously integrated with antimicrobials to ensure sustained antimicrobial protection. The antimicrobials employed range from synthetic drugs to herbal extracts and biologically or chemically active molecules. In a recent study, gelatin methacryloyl (GelMA), loaded with CHX, displayed remarkable antimicrobial potential in RET applications [204]. Derived from the modification of gelatin’s amine-containing side groups with methacrylamide and methacrylate groups, GelMA serves as a highly biocompatible matrix that facilitates favorable biological interactions. This novel GelMA hydrogel containing CHX exhibits a more prominent antibiofilm ability against *E. faecalis* compared to CHX and presents no obvious toxicity toward stem cells from human exfoliated deciduous teeth (SHEDs) [204]. In another study, GelMA hydrogels containing CHX or metronidazole were formulated and evaluated [205]. Both CHX@gGel and MTR@gGel systems were effective against *E. faecalis*, *S. mutans,* and *P. intermedia* in vitro, further highlighting the versatility and potential of antimicrobial-loaded hydrogels in endodontic therapy.

## 5. Conclusions and Future Perspectives

**Figure 7:**
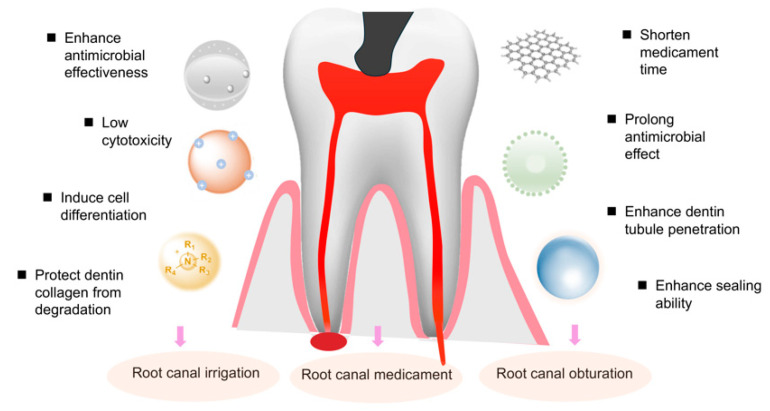
A schematic illustration of the potential applications and benefits of representative functional nanomaterials in the endodontic field.

The primary goal of treating endodontic infections is to eliminate biofilms from infected dentin tubules and to foster a conducive environment for the healing of apical tissues. Nanomaterials show great promise in addressing the challenges associated with the debridement, disinfection, and obturation of the intricate root canal system (Figure 7). This review compiles the most recent advances in employing nanomaterials for enhancing endodontic treatment procedures. However, it is important to note that many aspects remain underexplored.

Current studies predominantly evaluate the antimicrobial efficacy of novel materials against *E. faecalis* biofilms. However, taking into account the complex diversity of microbes existing in the root canal system, they may be overly limited. Research has largely focused on monospecies biofilms rather than multispecies or clinically relevant biofilms, which could provide a more accurate assessment of the antimicrobial capabilities of new compounds. While certain aspects like microbial viability and biofilm structure have received considerable attention, other important factors such as the penetration depth of nanoparticles, their ability to disrupt biofilm matrices, their antimicrobial mechanisms, and the potential for resistance development are less studied. Notably, the adaptation of bacteria to AgNPs has been extensively documented, though resistance is generally not considered likely [206].

A recognized limitation of the studies reviewed is their simplistic design, which predominantly focuses on antimicrobial effects. Materials developed for endodontic treatment should be multifaceted, not only possessing antimicrobial properties but also considering biocompatibility with surrounding cells, the physical properties of the root canal dentin, and potential systemic toxicity. Concerns about the cytotoxicity of nanoparticles, such as AgNPs, have been raised; their toxicity is influenced by their physical properties and dosage [207,208]. Furthermore, high doses of metal nanoparticles have been linked to systemic side effects like weight loss, tissue fibrosis, and increased oxidative stress [209,210,211]. In the case of intracanal medicament and root canal obturation materials, short-term and long-term biosafety evaluations of the novel agent should be performed before its implementation in clinical practice.

## Supplementary Materials

The following supporting information can be downloaded at: https://www.mdpi.com/article/10.3390/pharmaceutics16060759/s1, Figure S1. Transmission electron microscopic image of silver nanoparticle. (A) TEM image of AgNPs. Inset shows UV-VIS absorption spectrum of AgNPs in water. (B) TEM images of AgNPs@SiO_2_; Figure S2: Scanning electron microscopic image of TiO_2_ nanoparticle. (a) TEM image of TiO_2_ at 1000× magnification. (b) TEM image of TiO_2_ at 5000× magnification.
