# Supplementary material for: Nanoarchitectonics-Based Materials as a Promising Strategy in the Treatment of Endodontic Infections
Source: Pharmaceutics. 2024 Jun 4;16(6):759. doi: 10.3390/pharmaceutics16060759 (PMC11207628; doi:10.3390/pharmaceutics16060759)

Figure S1. Transmission electron microscopic image of silver nanoparticle.

Figure S1. Transmission electron microscopic image of silver nanoparticle. (A) TEM image of AgNPs. Inset shows UV-VIS absorption spectrum of AgNPs in water. (B) TEM images of AgNPs@SiO_2.

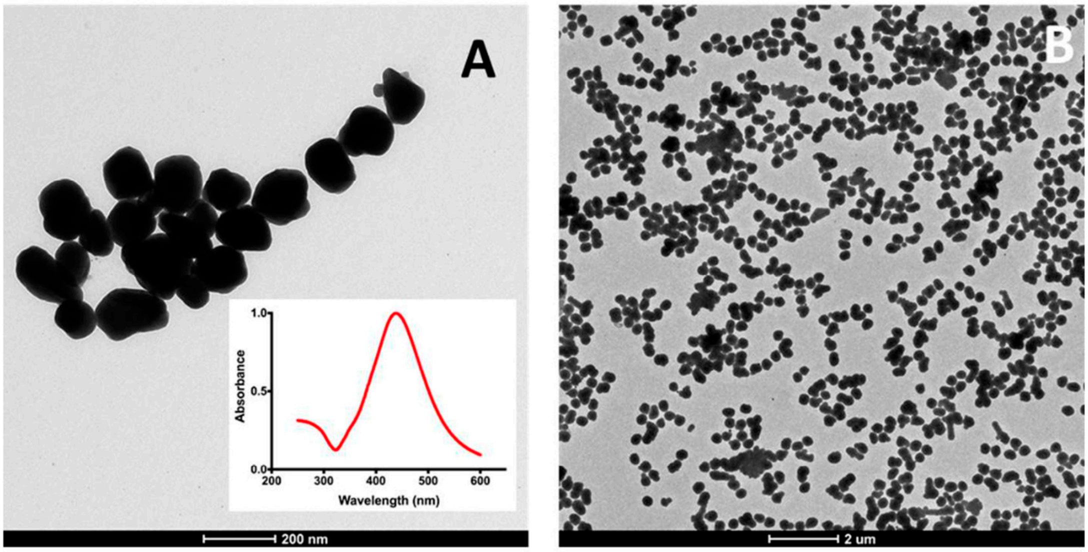

Figure S2: Scanning electron microscopic image of TiO_2 nanoparticle. (A) TEM im-age of TiO_2 at 1000× magnification. (B) TEM image of TiO_2 at 5000× magnification.

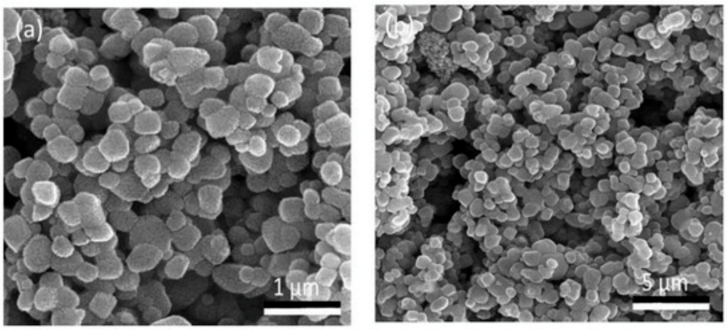

Supplement: Supplementary file 1 [file pharmaceutics-16-00759-s001.zip › pharmaceutics-2997841-supplementary.pdf]
